# Bone-Anchored Hearing Aid Effects on Vestibular Function: A Preliminary Report

**DOI:** 10.3390/audiolres14020033

**Published:** 2024-04-20

**Authors:** Federica Pollastri, Beatrice Giannoni, Vincenzo Marcelli, Giulia Spadavecchia, Rudi Pecci

**Affiliations:** 1 Unit of Audiology, Careggi University Hospital, Largo Brambilla 3, 50134 Florence, Italy; beatrice.giannoni@unifi.it (B.G.); giu.spada95@gmail.com (G.S.); peccirudi@gmail.com (R.P.); 2 Department of Neuroscience, Psychology, Drug’s Area and Child’s Health, University of Florence, 50134 Florence, Italy; 3 Neurotology Unit, Ospedale del Mare, 80147 Naples, Italy; vincenzo.marcelli@hotmail.it

**Keywords:** bone-anchored hearing aids, bone-conducted per-vibratory stimulus, skull vibration-induced nystagmus, skull vibration-induced nystagmus test

## Abstract

Objective: Cochlear receptors are sensitive to vibratory stimuli. Based on this sensibility, bone-anchored hearing aids have been introduced to correct unilateral or bilateral conductive or mixed hearing loss and unilateral deafness. The vestibular system is also sensitive to the vibratory stimulus and this type of response is used in clinics to test its functionality. Being aware of this double separated sensibility, we wondered whether bone vibration, which activates the acoustic receptors of patients with bone conduction aids, can also influence the functionality of the vestibular system. Methods: To this end, we recruited 12 patients with a bone-anchored hearing aid and evaluated their vestibular function with and without an activated vibratory acoustic device. Results: Our results show that the vibratory stimulus delivered by the bone conduction aid also reaches and stimulates the vestibular receptors; this stimulation is evidenced by the appearance or modification of some nystagmus findings during bedside vestibular testing. Despite this, none of these patients complained of dizziness or vertigo during prosthesis use. Nystagmus that appeared or changed during acoustic vibratory stimulation through the prosthesis was almost all predominantly horizontal, unidirectional with respect to gaze or body position, inhibited by fixation, and most often consistent with vestibular function tests indicating peripheral vestibular damage. Conclusions: The findings of sound-evoked nystagmus seem to indicate peripheral rather than central vestibular activation. The occurrence of some predominantly horizontal and high-frequency induced nystagmus seems to attribute the response mainly to the utricle and lateral semicircular canal.

## 1. Introduction

Bone-anchored hearing aids are emerging as one of the most effective prosthetic devices for correcting unilateral or bilateral conductive or mixed hearing losses for which conventional hearing aids are ineffective or contraindicated. Recently, their use has been extended to the restoration of hearing quality in cases of unilateral deafness. Bone-anchored hearing aids use the body’s natural ability to transmit sound through the bones. They process sound waves, converting them into vibrations that are anchored to a small titanium implant and transmitted to the inner ear, bypassing a pathological outer or middle ear or, in the case of unilateral hearing loss, stimulating the remaining cochlea [1].

Environmental sound sources transmitted to the inner ear via bone vibration may also result in simultaneous stimulation of vestibular receptors and/or consequent activation of central vestibular pathways. In 1935, von Bekesy first realized that vibration applied to the skull could induce an illusion of motion by stimulating vestibular receptors [2].

In 1973, Lucke demonstrated that applying a 100 Hz vibration to the mastoid of a subject with unilateral labyrinthine hypofunction produced a nystagmus with the fast phase directed to the healthy side [3].

Since then, the skull vibration-induced nystagmus test (SVINT) has been mainly used in clinical neuro-otology to detect vestibular peripheral asymmetries as a complementary or alternative test to thermal stimulation. In fact, under these conditions, a 100 Hz bone vibration applied to one of the mastoids immediately induces a predominantly horizontal nystagmus (skull vibration-induced nystagmus—SVIN), with the fast phase generally directed toward the healthy side. In fact, SVINT would act as a “vestibular Weber test” because, in the case of vestibular asymmetry, as in the case of auditory asymmetry, the bone vibration stimulation reaching the vestibular sensory apparatus of the dominant side produces a nystagmus response with a rapid phase that is directed towards that side [4,5,6,7].

In 1977, Young et al. first reported that primary afferents from the semicircular canals (SSCs) and otoliths of squirrel monkeys were both activated by bone-conducted vibration (BCV), laying the foundation for the pathophysiological explanation of SVIN [8].

Actually, SVIN has horizontal (98%), torsional (75%), and vertical (47%) components, suggesting a primary involvement of the horizontal semicircular canal (HSC) and utricle for the production of the horizontal component, the posterior and superior SCCs and/or sacculus for the vertical component, and the superior and posterior SCCs and/or otolith structures for the torsional component [6,9].

SVIN can also be a manifestation of central vestibular dysfunction, in which case it presents mainly as downbeat nystagmus, but sometimes also as horizontal nystagmus.

Another neurotological application that demonstrates the effect of bone vibration on the vestibular apparatus is that of bone-conducted vestibular evoked myogenic potentials (BC VEMPs). These are otolith-dependent reflexes produced by stimulating the ears with skull vibration and recorded from surface electrodes. Depending on where they are recorded from, they represent saccular (cervical VEMPs—cVEMPs) or utricular (ocular VEMPs—oVEMPs) functionality. Bone-conducted (and air-conducted—AC) VEMPs are also used in the diagnosis of superior semicircular canal dehiscence syndrome [10,11,12].

Although VEMPs are not an overly complicated clinical procedure, with standard evoked potential recording equipment, they cannot be considered as a bedside method as practical and rapid as would be desirable for basic clinical diagnostic routine.

Despite the above applications of vibratory stimulation to study the pathophysiology of the vestibular system, to the best of our knowledge, no investigative studies have been conducted and no application of potential vibratory stimulation of bone-anchored hearing prostheses has been performed.

The aim of our observational and retrospective pilot study was to investigate whether the use of a bone-anchored hearing prosthesis can produce any clinical objective or subjective effect on vestibular function due to vibratory stimuli induced by the active device.

## 2. Material and Methods

Our cohort is composed of 12 patients who underwent surgery with a bone-anchored hearing prosthesis (Ponto^®^ by Oticon Medical AB, Askim, Sweden) at the Audiology Unit of the Careggi University Hospital in Florence between 2015 and 2019. These were patients with bilateral (9) or unilateral (3) conductive or mixed hearing loss due to chronic phlogistic pathology or malformation (Table 1). None of the patients who underwent canal wall down and canal wall up tympanoplasty had a lateral semicircular canal fistula or labyrinthine erosion due to cholesteatoma at the time of surgery. Patients with otosclerosis who underwent stapedoplasty did not have any complications of perilymphatic fistula during surgery or sequelae of vertigo after surgery.

The pathologic condition was documented in all patients both objectively and by petrous bone CT scan.

The average bone and air pure tone audiometry (BC-AC PTA) thresholds at 500–1000–2000–4000 Hz at the implanted side, before surgery, were 46.625 dB and 83,166 dB, respectively.

Indication for the most appropriate processor to implant was assessed based on the company’s (Oticon Medical AB, Askim, Sweden^®^) reported Maximum Force Output (MFO) and calculated BC Pure Tone Audiometry (BC PTA) thresholds at 500–1000–2000–4000 Hz. Patients with a mean hearing loss of up to 45 dB received a bone-anchored hearing aid Ponto 3^®^ prosthesis; patients with a mean BC PTA between 45 and 55 dB received a Ponto 3 Power^®^ or Ponto Plus Power^®^ prosthesis; and patients with a BC PTA between 55 and 65 dB received a Ponto 3 Super Power^®^ prosthesis. Prior to surgery, all patients underwent the soft band trial to predict the potential prosthetic yield. The implantation technique used in all cases was Micro Invasive Ponto Surgery^®^ (MIPS).

The implanted side was the one with the worst AC, but also the one with the most ap-propriate BC threshold. When programming the prosthetic solution, the proposed mean BC threshold of 65 dB was never exceeded [13].

At the time we decided to perform the study under review, patients had already been operated on for an average of 27 months, with a minimum of 12 and a maximum of 60 months.

A general and specific history was taken, particularly regarding the presence of vestibular symptoms (dizziness, vertigo, postural disturbance), their nature (spontaneous or associated with causal factors such as movements, pressure changes, acoustic or visual stimuli). The vestibular examination was performed in a bedside modality, assessing conjugated ocular motility, frontal plane ocular static, visual-vestibular interaction using the vestibulo–ocular reflex (VOR) cancellation test (VCT) and the visually enhanced VOR test (VEVT), horizontal high-frequency dynamic VOR gain using the clinical head impulse test (cHIT), the possible presence of head shaking nystagmus (HSNy) by head shaking test (HST), and the symmetry of horizontal low-frequency dynamic VOR gain, assessed by comparing the number and amplitude of nystagmus beats when the patient’s head is slowly rotated without fixation.

SVINT was then performed under video-oculoscopy (VOS) using a 100 Hz bone vibrator, model Euroclinic^®^ VVS ED 500 (Medi-care Solutions, Euroclinic, Imola, Italy), placed and pressed on the mastoid process in line with the external acoustic meatus for 10 s, with three repetitions for each side.

When possible (depending on the patient’s level of cooperation and their cervical or visual conditions), patients without hearing aids were also tested for quantitative measurement of horizontal dynamic VOR gain using a video head impulse test (v-HIT) device (OtoSuite Vestibular Software Version 4.00 build 1286, Natus by Otometrics^®^, GN Otometrics A/S, Middleton, WI, USA). Horizontal dynamic VOR gain was considered asymmetric if the response difference between the two sides was greater than 0.20.

The VOS was then used to assess spontaneous and gaze-evoked nystagmus in the sitting position, spontaneous-positional nystagmus (in the supine, right, left and head-hanging positions) and positioning nystagmus (by performing right and left Dix-Hallpike test).

The presence and characteristics of nystagmus were first assessed with the inactive bone-anchored hearing prosthesis and then by sending some vibratory sound stimuli of different frequencies and intensities through the activated prosthesis to the inner ear. The prosthesis was connected by cable to a personal computer (PC) and, using the Genie Medical^®^ software (Genie Medical BAHS 2022.1.0 build 15.19.13, Oticon Medical AB), we used the BC in situ function to send the sound pulses. The vibratory sound stimulus was delivered for 15 s at an intensity of 35 dB for the frequency of 250 Hz and, in increasing order, at intensities of 40 and 60 dB for the frequencies of 500, 1000 and 2000 Hz. The vibratory sound intensities were always within the comfortable hearing range of the prosthesis for the specific frequency.

The possible presence and nature of vestibular signs manifested with and without vibratory acoustic stimulation delivered by the prosthesis were assessed and discussed by two experienced neuro-otologists (BG, RP) performing the test. Variation in spontaneous/positional and gaze-evoked nystagmus with stimulation was considered to be the appearance or amplitude/frequency modification of pre-existing basal findings.

If confirmed, the presence/absence of findings, the plane and direction of any nystagmus, and the appearance or change in amplitude were reported on a graph.

Under all test conditions, subjects were asked to report any occurrence of even modest symptoms. When present, subjective and objective findings were compared.

Similarly, when a nystagmus change occurred, patients were asked about the possible concurrent occurrence of subjective symptoms.

Comparison of vestibular findings in the absence and presence of vibroacoustic bone stimulation was possible only with respect to the presence of spontaneous, positional, positional, and gaze-evoked nystagmus. In fact, the results of VOR function tests with rapid head movements do not allow visualization of the vestibular response that may be evoked by stimulation through the prosthesis. However, the results of these latter assessments have been used to estimate VOR gain, the presence of asymmetry between the two vestibular hemi-systems, and/or evidence of central vestibular pathway involvement.

Overall patient satisfaction with the hearing benefit of the semi-implantable bone-anchored hearing prosthesis was assessed using a visual analogue scale (VAS) where ‘0’ represented no subjective functional outcome and ‘10’ represented a personal assessment of complete hearing recovery with the implanted prosthesis.

The results were reported in a database in which we recorded demographic (sex, age at surgery, age at visit) and anamnestic data (general and specific comorbidities), details of otological pathology (typology, laterality, type of hearing loss, BC PTA, AC PTA, vestibular symptoms pre-existing or present after surgery), implanted processor type, bedside neuro-otological examination findings, both with and without the device off and on, and VAS score before and after prosthesis activation.

All procedures performed in this study involving human participants were in accordance with the ethical standards of the institutional and/or national research committee and with the Helsinki Declaration of 1964 and its subsequent amendments or comparable ethical standards.

Informed consent was obtained from subjects prior to participation.

The entire study was approved by the local ethics committee of the Careggi University Hospital, Florence, Italy.

## 3. Results

This study included seven women and five men. At the time of surgery, the mean age of the subjects was 65.58 years (women: 59.3 years; men: 77.2 years), with a minimum of 32 and a maximum of 81 years. At the time of assessment, the mean age was 68.3 years (women: 61.1 years; men: 77.4 years), with a minimum of 36 and a maximum of 83 years. The time interval between the insertion of the hearing aid and the time of evaluation for this study averaged 2.75 years, with a minimum of 1 and a maximum of 5.

In Table 2, we report in detail the age of every patient at the time of bone-anchored hearing implant surgery and at the time of the neuro-otological evaluation, as well as the time interval between the two events.

At the time of assessment, pure-tone audiometry revealed bilateral mixed hearing loss in eight cases (66.8%), unilateral mixed hearing loss in one case (the latter being the only hearing ear) (8.3%), bilateral hearing loss of different types (mixed in one ear and sensorineural in the other due to presbycusis) in one case (8.3%), and bilateral pure conductive hearing loss in the remaining two subjects (16.6%).

Right and left AC PTA mean values were 78.65 dB (min. 36.25; max. 110) and 74.73 dB (min. 31.25; max. 101.25), respectively. Bone conduction PTA mean values were 43.40 dB (min. 18.75; max. 68.75) and 42.98 dB (min. 25.0; max. 68.75) for the right and left ear, respectively. These data are detailed in Table 3.

Seven patients (58.3%) had a history of cardiovascular risk factors (hypertension, hypercholesterolemia, hyperuricemia, diabetes, atrial fibrillation, and smoking), one of whom also had multiple sclerosis. Two patients (16.7%) presented with syndromic pathology (Turner’s and Digeorge syndromes); three of the case study subjects (25%) had no significant disease other than ear pathology.

The mean pre- and post-implantation VAS scores were 2.75 and 8.5 points, respectively. Comparing the VAS results before and after surgery, the mean subjective hearing improvement was 5.75 points.

Eleven of 12 patients (92%) had no history of vestibular dysfunction (vertigo, dizziness, or vertigo) or migraine-like headaches prior to surgery. The remaining patient, who had multiple sclerosis, complained of a non-specific, long-standing disequilibrium. None of the subjects experienced any vestibular symptoms at either short or long distances after prosthesis implantation.

When investigating possible vestibular findings, we started from a “baseline” condition, i.e., in the absence of any bone stimulation by the prosthesis.

In this condition, bedside ocular motility testing was relatively normal in all patients except one in whom slow tracking eye movements were interrupted by saccades due to reduced gain in both the horizontal and vertical planes. None of the patients had intrinsically pathological saccadic eye movements, oblique deviation, or altered VCT or VEVT.

Five out of 12 (41.7%) subjects had a negative HST and 7/12 (58.3%) patients had horizontal HSNy, the latter revealing a mid-frequency vestibular imbalance between the two sides. None of the patients had vertical HSNy.

Clinical HIT revealed symmetrical high-frequency horizontal VOR gain in all patients. Such symmetry was due to presumed normal bilateral gain in 7/12 patients (58.3%) with a negative test and to bilateral vestibular hypofunction in the remaining 5 (41.7%) with a bilateral positive test.

The vHIT could not be performed in 5/12 cases (41.7%) due to poor compliance or patient cervical rigidity; in the remaining 7 subjects, this test recorded symmetric VOR gain values in 3 (25%) and indicated gain asymmetry in the remaining 4 patients (33.3%).

Medium- and high-frequency horizontal angular VOR functionality test results with prosthesis off are reported in Table 4.

In the absence of vibroacoustic stimulation through the prosthesis, 5/12 patients (41.7%) showed no spontaneous/positional and gaze-evoked nystagmus. Conversely, 7 of 12 (58.3%) had some nystagmus findings indicative of vestibular asymmetry. Such spontaneous/positional nystagmus was mainly horizontal in 6 of 7 patients (85.7%), directed to the better ear in 4/6 cases and to the worse ear in 2/6 cases. Only 1/7 patients showed a low-amplitude, multipositional, upward nystagmus.

Of the five patients with no signs at baseline, one later showed nystagmus directed to the pathologic ear when vibroacoustic stimulation was applied.

Without vibratory stimulation, VOR function tests and nystagmus findings together indicated vestibular asymmetry in 10 of 12 cases (83.3%).

Because they were elicited by a stimulus closer to the study conditions, the SVINT results were considered separately.

Prior to prosthesis activation, bone vibration at 100 Hz applied bilaterally and alternately to the mastoid produced predominantly horizontal nystagmus in 9 of 12 patients (75%), indicative of vestibular imbalance; the test was negative in the remaining 3 subjects (25%).

At baseline, all nine SVINT-positive subjects also showed evidence of vestibular asymmetry as evidenced by the simultaneous presence of spontaneous/positional and lateral gaze-induced nystagmus (6/12 patients, 50%) and/or alteration of angular VOR function tests (9/12 patients, 75%). In the nine SVINT-positive subjects, the SVIN beat to the healthy side in three, changed its fast phase direction according to the stimulated mastoid in three, and was directed to the pathologic side in the remaining three (Table 5).

The above results show that only 2 (16.7%) of the 12 patients studied had no evidence of pre-existing vestibular asymmetry at baseline.

As mentioned in Section 2, the comparison between basal conditions and bone vibration stimuli delivered by the activated prosthesis could only be made for spontaneous/positional and gaze-evoked nystagmus findings.

By delivering the vibroacoustic stimulus through the prosthesis, we observed a change in nystagmus findings in 8 of 12 patients (66.7%); in the remaining 4 (33.3%), the prosthesis delivered stimulus did not lead to any change.

Of the eight patients who showed a per-stimulus change in nystagmus, seven already had vestibular asymmetry/imbalance as evidenced by the presence of spontaneous/positional nystagmus in basal conditions and/or (at least) one positive functionality test. Of these seven patients, six had horizontal spontaneous/positional nystagmus and one had oblique upbeat nystagmus.

Although increased in amplitude and angular velocity, the per-stimulus findings were not different in direction and plane from baseline.

The only patient with a de novo finding on stimulation had a predominantly horizontal nystagmus.

The condition in which we noticed the most nystagmus variation was left gaze, followed by right gaze, head up, sitting, supine, left side and right side. We found a progressive increase in nystagmus variations in proportion to the increase in both the frequency and intensity of the stimulation provided by the bone conduction aid.

Only one patient, who did not show any nystagmus during the neuro-otological examination in baseline conditions, showed the appearance of findings during stimulation. In this case, nystagmus was mainly found on the right lateral side and with the gaze right. In this case, most of the variations of signs have been noted at the frequency of 1000 Hz (40 dB) and 2000 Hz (40 dB).

In Table 6, we report, in detail, the situation of spontaneous/positional and gaze-evoked nystagmus with the device off and the variations emerged under vibroacoustic stimulation, associated with VOR functionality tests results.

The vibroacoustic stimulation delivered through the prosthesis that produced the greatest number of changes in nystagmus findings compared to the no-stimulus condition was 2000 Hz, followed by 1000 Hz and finally 250 and 500 Hz.

The reappearance or change in findings at the arrival of vibroacoustic stimulation always occurred during the application of the stimulus. In no case did we detect additional findings or changes to those that existed when the stimulation ceased.

One patient was completely negative to all tests and another one, as just mentioned, had nystagmus only under stimuli.

Of the patients without per-stimulatory nystagmus findings, one still had a positive SVINT and HST, while two had a positive HST associated with asymmetry on v-HIT, in basal conditions.

Two patients showed evidence of asymmetry on all VOR function tests as well as nystagmus findings, both with the prosthesis off and on.

In four cases, spontaneous/positional and gaze-evoked nystagmus was present both with the device off and on, in addition to a positive HST and SVINT. The last patient emphasized the presence of nystagmus with and without vibratory stimulation, but the VOR function tests were all normal.

Even when changes in nystagmus findings were clearly evident, none of the patients ever experienced any kind of vestibular disturbance under stimulation.

## 4. Discussion

Patient demographics are consistent with those reported in the literature for patient cohorts undergoing BAHA surgery in general [14]. In addition, the indication for surgery did not differ from what is considered appropriate [15,16]. Therefore, neither demographic data nor the underlying pathological condition can be held responsible for any differences in outcome with this type of prosthesis in our study cohort.

However, as an effect of the random selection of consecutive patients, there is a prevalence of bilateral hearing loss in our series and no case of unilateral deafness.

The implanted processors available on the market were evenly distributed among the patients in terms of power. In particular, the eight subjects who presented appearance/variation in findings under stimulation had indifferent low-, medium- and high- power processors. In no way, therefore, should the greater or lesser power of bone stimulation have affected the generation of any stimulation of the vestibular apparatus.

The Visual Analog Scale results showed a high degree of satisfaction with the prosthesis and a medium degree of subjective hearing recovery in all patients. The MIPS technique used for implantation also contributed to a good acceptance of the surgical solution [17]. If the application of a bone prosthesis had disturbed vestibular function, the degree of acceptance and satisfaction with the implanted device would not have been good on average.

After surgery and up to the time of our evaluation, none of the patients reported symptoms of dizziness, imbalance, postural disturbance, or other discomfort suggestive of vestibular dysfunction. This finding might suggest that the power of the acoustic bone stimulation normally delivered by the prosthesis as needed according to the tonal audiogram was not able to disturb vestibular function.

Even during the performance of functionality tests and assessment of spontaneous/positional and gaze-evoked nystagmus, the latter both with the device off and on, none of the subjects ever reported the occurrence of vestibular symptoms of any type or degree.

Taking into account spontaneous findings and VOR function tests, despite the absence of subjective symptoms, the baseline examination still showed objective signs of imbalance between the two vestibular hemi-systems in a large percentage of patients (9/12—75%). Since the horizontal angular VOR function was asymmetric in most of the patients (75%) before stimulation by the prosthesis, it can be concluded that the asymmetry was determined by the underlying otologic pathology; this should also mean that the signal imbalance was subjectively compensated, since the patients did not report any acute or subacute symptoms. This asymmetry was most pronounced for the mid/high-frequency VOR stimuli at 2 Hz (HST, 7/9, 77.7%) and to a lesser extent also at 6 Hz (HIT, 4/9 pts, 44.4%).

The sensitivity of SVINT to detect imbalance was very high and was positive in most of the asymptomatic patients (9/12, 75%). This sensitivity is in line with what has been reported in the literature for the test [18].

In the whole series, the absence of obvious alterations in ocular motricity, ocular statics in the frontal plane and VCT allowed us to exclude important alterations in the central visual-vestibular oculomotor circuits. Moreover, most of patients did not even have obvious central lesions on CT.

We could speculate that the imbalance in the vestibular signal prior to stimulation by the prosthesis could reasonably be considered primarily or secondarily of peripheral origin for a number of reasons, namely: (a) all patients had pathology of the ear, which could potentially also partially involve its inner part; (b) signs never suggested a definite central involvement (such as nystagmus not inhibited by fixation, nystagmus and/or nystagmus with paradoxical amplitude and frequency or direction changing with gaze or position; (c) none but one patient had remote or recent symptoms suggestive of central vestibular or neurological disorders. Furthermore, newly appearing findings or changes in already-present signs evoked by stimulation were always strictly per-vibrational, i.e., they appeared and ceased by administering and removing the stimulus, respectively. This type of response to vibratory stimulation is considered typical in cases of signal imbalance at the labyrinthine level [6,7]. The absence of signs at the cessation of the stimulus, even if this has been relatively prolonged, should in fact indicate the lack of activation of the velocity storage mechanism and therefore the involvement of brainstem vestibular structures.

When examining the spontaneous/evoked positional nystagmus findings when bone sound stimulation was delivered through the prosthesis, the appearance or increase in amplitude of one or more of these findings occurred in two-thirds of the cases. The accentuated or increased nystagmus following acoustic/vibratory stimulation occurred in the horizontal plane in most cases, and very rarely had a small vertical component. Only one patient was positive for the presence of vertical nystagmus in some study positions, both in basal conditions and, more obviously, under stimulation.

These data confirm what Zamora et al. observed in 2018 with respect to the vestibular response to mastoid vibratory stimulation, namely that neural activation is mainly related to the lateral semicircular canal and utricle receptors rather than those of the vertical semicircular canals and the saccule [19].

From the literature on the vestibular effects of transcutaneous vibratory stimulation it is known that the appearance or modification of nystagmus under bone vibration reveals the presence of an asymmetry between the two vestibular hemi-systems or is the effect of an increase in the asymmetry itself. In most cases, the per-vibratory nystagmus with its fast phase is directed to the functionally better side [6,7,20]. This is due to a relatively reduced per-stimulatory peripheral afferent signal coming from the functionally worse labyrinth. Conversely, in the case of labyrinthine asymmetry, cranial vibration leads to an increase in neural signals from the better side, resulting in an imbalance between the two vestibular nuclei [6,7,18].

In certain conditions, such as hydropic vestibulopathy or in the recovery phase of unilateral or asymmetric deficient vestibulopathy, a vibration-induced nystagmus may be directed to the opposite (primitively worse) side. In fact, a situation of imbalance in favor of the deficient hemi-system can be due both to an irritative hypertonus of one of the two hemi-systems or to an “overcompensation” during the natural processes of neural rebalancing that follow a unilateral deficit vestibulopathy. When this occurs, it occurs sometime after the primary event and can be maintained for a significant period of time [21,22,23].

In our case series, the direction of the rapid phase of spontaneous/positional nystagmus induced or enhanced by bone vibration stimulation was, in most cases (6/8), toward the ear that was functionally worse from an auditory point of view, i.e., hypothetically, the worse in terms of vestibular function. In only one out of eight cases was the positional nystagmus directed to the better side, and in one patient the nystagmus finding did not clearly indicate a specific side.

Since the basic otologic pathology of the patients in our series was mostly that of chronic otitis or its sequelae, and never that of a hydropic condition, the presence of a nystagmus directed to the probably deficient side can only be explained by a latent imbalance of the two vestibular hemi-systems in favor of the worse side, due to compensatory phenomena in different evolutionary stages. The spontaneous positional findings evoked or enhanced under stimulation could represent the accentuation of this imbalance.

Comparing SVINT nystagmus findings (therefore evoked by a 100 Hz bone vibration) with vibroacoustic stimulation-induced nystagmus at 250–500–1000–2000 Hz, it appears that subjects who had the appearance/modification of spontaneous/evoked positional nystagmus also had a positive SVINT. However, the opposite is not true; in fact, three subjects had SVIN as the only finding induced by vibratory stimulation.

From the above, it could be argued that there is a vestibular sensitivity to vibratory stress, although it varies with stimulus frequency. In our experience, it was more common to have a high-frequency per-vibratory acoustic induced/increased nystagmus when this finding was also present at lower frequencies (SVIN). The difference in frequency response may be related to the type of receptors/neurons being recruited [8,12,19,20].

Also in terms of quality, the SVINT findings were at most consistent with those elicited by prosthesis activation, confirming that the latter should also be of peripheral origin.

Only one patient, who showed no sign with the device off, later noted nystagmus during stimulation. In the latter case, the per-vibratory nystagmus occurred with stimulation at all frequencies tested except 500 Hz. Again, this patient did not report any symptoms after the procedure nor any subjective vestibular disturbance during stimulation. Therefore, it could be observed that an operative bone prosthesis can effectively stress the vestibular organ, revealing a latent tiny functional asymmetry only by means of vibratory acoustic stimuli. However, even in this condition, the asymmetry is so small that the patient does not perceive it.

From a quantitative and qualitative analysis of gaze-evoked and spontaneous/positional findings under vibroacoustic stimulation, it could be observed that these signs increased with increasing stimulation frequency. In particular, most of the changes in the findings were observed with the 2000 Hz vibroacoustic stimulus.

From experimental studies of bone stimulation reported in the literature (Curthoys 2016), it is known that a pure tone with a frequency between 100 and 2000 Hz activates the irregular primary afferent neurons of the vestibular macula, even at a low threshold. These cells, when activated, show a phase-locked activity of the action potential at the single stimulus cycles, similar to that found in auditory afferents [24,25].

On the other hand, afferent neurons from the semicircular canals are preferentially activated by stimulation within 200 Hz and do not show higher frequency discharge. As the frequency decreases to about 100 Hz, the irregular neurons of the horizontal and anterior semicircular canals show phase-locked activation [7,26].

Since, in our experience, the stimulus frequency that produced the greatest variation in nystagmus findings was that of 2000 Hz, it might be hypothesized that the structures that produce this vestibular response correspond to the macular organs. The vibratory stimulus at these frequencies could act by highlighting a primitive or secondary latent imbalance at the level of the afferent utricular pathways, as can be seen by the appearance of a predominantly horizontal nystagmus. The hypothesis of a macular origin of these findings evoked by an acoustic-vibratory stimulus could also be supported by their low amplitude.

Since, in our experience, the stimulus frequency that produced the greatest variation in nystagmus findings was that of 2000 Hz, it can be hypothesized that the structures that produce this vestibular response correspond to the macular organs. The vibratory stimulus at these frequencies would act by highlighting a primitive or secondary latent imbalance at the level of the afferent utricular pathways, as can be seen by the appearance of a predominantly horizontal nystagmus. The hypothesis of a macular origin of these findings evoked by an acoustic-vibratory stimulus is also supported by their low amplitude.

Conversely, responses obtained with SVINT (100 Hz bone vibration) and with bone vibratory acoustic stimulation at 250 Hz (delivered only at the intensity of 35 dB) could be due to simultaneous activation of both the utricular macula and the lateral semicircular canal. The bilateral stimulation of these receptors would lead to a horizontal per-vibratory nystagmus in the case of a latent asymmetry between the two vestibular hemi-systems. In fact, in the case of functional symmetry between the two hemi-systems, SVIN is not generated because the stimulus determines a bilateral and symmetrical activation of the receptors and, therefore, a balanced and nuclear equivalent input that does not produce any eye movement.

Our results seem to be partially different from what Curthoys stated in 2021. In fact, the recording of vibratory afferent stimulus discharge in guinea pigs with a single remaining labyrinth function showed that the most effective stimulus occurs at 100 Hz. According to the author’s findings, this frequency would activate the irregular discharging neurons of the semicircular canals and otolith organs. These neurons exhibit firing activity with a variable, generally small, interval between action potentials in the absence of stimulus, called resting discharge. Irregular neurons are present at the level of the calyx endings on type 1 receptors of the highest part of the ampullary crest and the striola of both maculae. When the frequency of the vibratory stimulus exceeds 100 Hz, the amplitude of the cranial vibration would decrease and consequently the displacement of the endolymphatic fluids would be less intense, causing a deflection of the stereocilia not sufficient to elicit an obvious response [20]. Certainly, our data on that are still small and need validation with larger numbers as well as being based on clinical observations and not experimental data.

Among the patients studied, there are also two in whom the vibratory stimulus nystagmus response pattern also showed vertical components. In these subjects, this particular response was observed mainly at higher stimulus frequencies. A slight latent asymmetry of the afferent saccular pathways could be hypothesized to explain this finding. Moreover, neither of these two patients had any other clinical or neuroradiological findings indicative of possible central vestibular dysfunction, so to explain their observation, the only reasoning we could come up with was that of an involvement of a peripheral receptor whose lesion is capable of giving rise to a nystagmus with vertical components.

Finally, we found the case of a patient in whom the vibroacoustic stimulus caused the onset of a nystagmus pattern suspected of central vestibular imbalance (up beating nystagmus with a small oblique component to the left). This sign is also described for the 100 Hz vibratory stimulus [7].

As our study is currently retrospective and newly designed, we did not have objective data on the semeiological and clinical vestibular situation of our patients before surgery and therefore could not compare it with the basal condition after surgery or even with that under stimulation. However, assuming a preoperative functional asymmetry in favor of the better hearing side, the time elapsed between surgery and our evaluation may have been sufficient to generate the static/dynamic vestibular compensation mechanisms capable of producing the “overcompensation” type functional imbalance (toward the initially worse side) that we observed.

A significant limitation of our study is that the sample of patients analyzed is quite small; therefore, it did not allow the application of statistical tests capable of providing certainly significant results. On the other hand, although the application of bone-anchored hearing prostheses is increasingly recommended and put in place, it is still true that the number of eligible patients could be reduced because the cases suitable for this type of prosthesis are fewer in number than those who can benefit from the traditional air conduction prosthesis and that quite a few patients refuse surgery or a device, albeit partially implanted in the skull bone and/or and the cosmetic encumbrance of the prosthesis.

In addition, our study was an observational and pilot study in which we attempted to verify whether vibroacoustic stimulation through a BAHA prosthesis could also produce symptoms and/or signs of vestibular activation. To achieve this, we had only objectively evaluated the appearance and nature of the findings that may be produced by this stimulus, using the bedside method. Therefore, in this study, we did not record nystagmus eye movements and thus did not quantitatively evaluate their extent.

Since our study seems to show some potentially useful results, we believe that future studies would need to increase the number of patients and include a flexible method that allows vibroacoustic stimulation and video and oculographic recording to be performed together without mutual interference.

## 5. Conclusions

The studies carried out on the effects of bone vibration stimulation have shown that this type of stress can also affect the activity of the vestibular apparatus, so that this type of stimulus applied to the skull is used to detect or characterize some neuro-otological pathologies.

Our pilot study seems to show that implantation of a bone-anchored hearing device is not associated with the onset of any perceptible vestibular dysfunction. Therefore, the success of the surgical prosthesis should not be compromised by the onset of potentially very disabling vestibular side effects.

However, in our study, we found that vibratory stimulation through the prosthesis can determine the appearance or modification of some vestibular findings, suggesting the influence of vibroacoustic stimulation on the vestibular-oculomotor reflex, both of canalar and macular origin, especially because of peripheral organ involvement. The per-vibratory objective changes occurred mainly at a high frequency, the latter finding leading to the hypothesis of their predominantly utricular origin. Also the low amplitude of the signs and the plane on which these small nystagmus are highlighted seem to indicate such an origin.

Despite having observed objective vestibular findings due to vibroacoustic stimulation, the appearance of any subjective disturbance in any patient has never been detected.

As a whole, the data obtained in our study do not currently allow us to consider a patient’s vestibular status as a contraindication to implantation of a BAHA prosthesis, nor to consider vestibular dysfunction as a possible postoperative side effect.

At the moment, however, ours is a preliminary and clinical study whose results need to be validated with further studies, possibly including a larger number of patients, recording and quantifying any nystagmus findings, and performing vestibular function testing just before surgery and postoperatively at fixed and equal cadences for all patients.

## Figures and Tables

**Table 1:** Outer and middle ear pathologies leading to BAHA implantation.

Outer/Middle Ear Pathology	Number of Patients (tot. 12)
Outcomes of bilateral chronic otitis undergoing bilateral canal wall down mastoidectomy	4
Outcomes of unilateral chronic otitis undergoing unilateral canal wall down mastoidectomy	2
Outcomes of bilateral chronic otitis undergoing bilateral canal wall up mastoidectomy	1
Bilateral recurrent otitis	2
Outcomes of bilateral otosclerosis undergoing bilateral stapedoplasty with poor hearing results	1
Tympanic perforation	1
Bilateral atresia auris	1

**Table 2:** Age of patients at the time of bone-anchored hearing implant surgery and neuro-otological evaluation, and time interval between the two events (all values are expressed in years).

ID	Sex	Age at Surgery	Age at Neuro-Otological Evaluation	Time Interval
1	F	48	52	4
2	F	32	36	4
3	M	80	83	3
4	F	71	74	3
5	F	40	42	2
6	M	77	78	1
7	M	81	82	1
8	M	71	72	1
9	F	76	77	1
10	F	70	73	3
11	M	72	77	5
12	F	69	74	5
Tot. 12 pts average		65.58	68.3	2.75
Tot. 12 pts minimum		32	36	1
Tot. 12 pts maximum		81	83	5

**Table 3:** Details on patient’s outer/middle ear pathology, bilateral air and bone conduction thresholds and the side chosen for the bone-anchored prosthesis implant All data are reported in dB HL. (Acronyms: PTA = pure tone average, AC = air conduction, BC = bone conduction, CWD = canal wall down, SPL = stapedoplasty, TM = tympanic membrane, and CWU = canal wall up).

ID	External/Middle Ear Pathology	Right Ear AC PTA	Left Ear AC PTA	Right Ear BC PTA	Left Ear BC PTA	Implanted Side
1	bilateral recurrent otitis	36.25	66.25	18.75	25.00	left
2	bilateral CWD mastoidectomy	73.75	101.25	52.50	58.75	left
3	bilateral CWD mastoidectomy	61.25	90.50	32.50	35.00	left
4	bilateral CWD mastoidectomy	76.25	86.25	53.75	56.25	left
5	bilateral atresia auris	70.00	78.75	30.00	30.00	left
6	unilateral CWD mastoidectomy/ contralateral recurrent otitis	110.0	72.50	undetectable	60.00	left
7	bilateral SPL	88.75	93.75	68.75	68.75	left
8	bilateral CWD mastoidectomy	82.50	72.50	38.75	33.75	right
9	TM perforation	81.25	31.25	43.75	26.25	right
10	bilateral CWU mastoidectomy	56.25	68.75	38.75	45.00	left
11	bilateral chronic otitis media	95	100.0	53.75	52.00	left
12	unilateral CWD mastoidectomy/ contralateral recurrent otitis	76.25	35.00	46.25	25.00	right
tot 12 pts average		75.63	74.73	43.40	42.98	
tot 12 pts minimum		36.25	31.25	18.75	25.00	
tot 12 pts maximum		110.0	101.25	68.75	68.75	

**Table 4:** Medium- and high-frequency horizontal angular VOR functionality test with prosthesis Off.

ID	HST	cHIT	Horizontal HF VOR Gain to the Right (v-HIT)	Horizontal HF VOR Gain to the Left (v-HIT)
1	negative	negative	-	-
2	positive	negative	0.95	0.82
3	negative	bilaterally positive	0.46	0.83
4	negative	negative	0.91	0.63
5	negative	negative	-	-
6	positive	bilaterally positive	1.26	0.62
7	positive	negative	0.87	0.76
8	positive	bilaterally positive	-	-
9	positive	negative	-	-
10	positive	bilaterally positive	0.72	0.48
11	positive	bilaterally positive	-	-
12	negative	negative	0.89	0.93

**Table 5:** Baseline conditions SVINT, spontaneous/positional, gaze-evoked nystagmus and VOR functionality test results.

ID	SVINT	Spontaneous/Positional and Gaze-Evoked Nystagmus	VOR Functionality Tests
1	negative	absent	negative
2	positive	absent	positive
3	positive	absent	positive
4	positive	absent	positive
5	negative	absent	negative
6	positive	present	positive
7	positive	present	positive
8	positive	present	positive
9	positive	present	positive
10	positive	present	positive
11	positive	present	positive
12	negative	present	negative

**Table 6:** Per-stimulatory spontaneous/positional and gaze-evoked nystagmus variations compared with basal conditions signs and with VOR functionality test results.

	Spontaneous/Positional and Gaze Evoked Nystagmus (Device Off)	Spontaneous/Positional and Gaze-Evoked Nystagmus Variation (Device On)	SVINT	HST	c-HIT	v-HIT
**1**	no	yes	negative	negative	negative	-
**2**	no	no	positive	positive	negative	symmetrical
**3**	no	no	positive	negative	bilaterally positive	asymmetrical
**4**	no	no	positive	negative	negative	asymmetrical
**5**	no	no	negative	negative	negative	-
**6**	yes	yes	positive	positive	bilaterally positive	asymmetrical
**7**	yes	yes	positive	positive	negative	symmetrical
**8**	yes	yes	positive	positive	bilaterally positive	-
**9**	yes	yes	positive	positive	negative	-
**10**	yes	yes	positive	positive	bilaterally positive	asymmetrical
**11**	yes	yes	positive	positive	bilaterally positive	-
**12**	yes	yes	negative	negative	negative	symmetrical

## Data Availability

No new data were created or analyzed in this study. Data sharing is not applicable to this article.

## References

[1] Westerkull P. (2011). The Ponto bone-anchored hearing system. Adv. Otorhinolaryngol..

[2] Von-Bekesy G. (1935). Uber akustishe Reizung des Vestibularapparates. Arch. Ges. Physiol..

[3] Lucke K. (1973). A vibratory stimulus of 100 Hz for provoking pathological nystagmus. Z. Laryngol. Rhinol. Otol..

[4] Hamann K.F., Schuster E.M. (1999). Vibration-induced nystagmus—A sign of unilateral vestibular deficit. ORL J. Otorhinolaryngol. Relat. Spec..

[5] Dumas G., Michel J., Lavieille J.P., Charachon R., Ouedraogo E. (1999). Clinical value of the cranial vibratory test: Results of a 3D analysis of nystagmus. J. Fr. ORL.

[6] Dumas G., Perrin P., Schmerber S. (2008). Nystagmus induced by high frequency vibrations of the skull in total unilateral peripheral vestibular lesions. Acta Otolaryngol..

[7] Dumas G., Curthoys I.S., Lion A., Perrin P., Schmerber S. (2017). The skull vibration-induced nystagmus test of vestibular function—A review. Front. Neurol..

[8] Young E.D., Fernández C., Goldberg J.M. (1977). Responses of squirrel monkey vestibular neurons to audio-frequency sound and head vibration. Acta Otolaryngol..

[9] Dumas G., De Waele C., Hamann K.F., Cohen B., Negrevergne M., Ulmer E., Schmerber S. (2007). Le test vibratoire osseux vestibulaire Skull vibration induced nystagmus test. Ann. Otolaryngol. Chir. Cervicofac..

[10] Kheradmand A., Zee D.S. (2012). The bedside examination of the vestibulo-ocular reflex (VOR): An update. Rev. Neurol..

[11] Rosengren S.M., Colebatch J.G., Young A.S., Govender S., Welgampola M.S. (2019). Vestibular evoked myogenic potentials in practice: Methods, pitfalls and clinical applications. Clin. Neurophysiol. Pract..

[12] Curthoys I.S., Grant J.W., Pastras C.J., Fröhlich L., Brown D.J. (2021). Similarities and Differences between Vestibular and Cochlear Systems—A Review of Clinical and Physiological Evidence. Front. Neurosci..

[13] Snik A.F.M., Bosman A.J., Mylanus E.A.M., Cremers C.W.R.J. (2004). Candidacy for the bone-anchored hearing aid. Audiol. Neurotol..

[14] de Wolf M.J., Hol M.K., Mylanus E.A., Snik A.F., Cremers C.W. (2011). Benefit and quality of life after bone-anchored hearing aid fitting in children with unilateral or bilateral hearing impairment. Arch. Otolaryngol. Head Neck Surg..

[15] Ghossaini S.N., Roehm P.C. (2019). Osseointegrated auditory devices: Bone-anchored hearing aid and PONTO. Otolaryngol. Clin. N. Am..

[16] Colquitt J.L., Loveman E., Baguley D.M., Mitchell T.E., Sheehan P.Z., Harris P., Proops D.W., Jones J., Clegg Aj Welch K. (2011). Bone-anchored hearing aids for people with bilateral hearing impairment: A systematic review. Clin. Otolaryngol..

[17] Di Giustino F., Vannucchi P., Pecci R., Mengucci A., Santimone R., Giannoni B. (2018). Bone-anchored hearing implant surgery: Our experience with linear incision and punch techniques. Acta Otorhinolaryngol. Ital..

[18] Curthoys I.S. (2021). The Neural Basis of Skull Vibration Induced Nystagmus (SVIN). Audiol. Res..

[19] Zamora E.G., Araùjo P.E.S., Guillén V.P., Gamarra M.F.V., Ferrer V.F., Rauch M.C., Garrigues H.P. (2018). Parameters of skull vibration-induced nystagmus in normal subjects. Eur. Arch. Oto-Rhino-Laryngol..

[20] Curthoys I.S. (2010). A critical review of the neurophysiological evidence underlying clinical vestibular testing using sound, vibration and galvanic stimuli. Clin. Neurophysiol..

[21] Manzari L., Burgess A.M., MacDougall H.G., Bradshaw A.P., Curthoys I.S. (2011). Rapid fluctuations in dynamic semicircular canal function in early Ménière’s disease. Eur. Arch. Otorhinolaryngol..

[22] Bance M., Mai M., Tomlinson D., Rutka J. (1991). The changing direction of nystagmus in acute Menière’s disease: Pathophysiological implications. Laryngoscope.

[23] McClure J.A., Copp J.C., Lycett P. (1981). Recovery nystagmus in Ménière’s disease. Laryngoscope.

[24] Curthoys I.S., Vulovic V., Burgess A.M., Sokolic L., Goonetilleke S.C. (2016). The response of guinea pig primary utricular and saccular irregular neurons to bone-conducted vibration (BCV) and air-conducted sound (ACS). Hear. Res..

[25] Rose J.E., Brugge J.F., Anderson D.J., Hind J.E. (1967). Phase-locked response to low-frequency tones in single auditory nerve fibers of the squirrel monkey. J. Neurophysiol..

[26] Curthoys I.S., Kim J., McPhedran S.K., Camp A.J. (2006). Bone conducted vibration selectively activates irregular primary otolithic vestibular neurons in the guinea pig. Exp. Brain Res..

